# Identification of the *Arabidopsis REDUCED DORMANCY 2* Gene Uncovers a Role for the Polymerase Associated Factor 1 Complex in Seed Dormancy

**DOI:** 10.1371/journal.pone.0022241

**Published:** 2011-07-25

**Authors:** Yongxiu Liu, Regina Geyer, Martijn van Zanten, Annaick Carles, Yong Li, Anja Hörold, Steven van Nocker, Wim J. J. Soppe

**Affiliations:** 1 Department of Plant Breeding and Genetics, Max Planck Institute for Plant Breeding Research, Cologne, Germany; 2 Beijing Botanical Garden, Institute of Botany, Beijing, China; 3 Freiburg Initiative for Systems Biology, Center for Biological Systems Analysis, University of Freiburg, Freiburg, Germany; 4 Department of Horticulture, Michigan State University, East Lansing, Michigan, United States of America; Michigan State University, United States of America

## Abstract

The life of a plant is characterized by major phase transitions. This includes the agriculturally important transitions from seed to seedling (germination) and from vegetative to generative growth (flowering induction). In many plant species, including *Arabidopsis thaliana*, freshly harvested seeds are dormant and incapable of germinating. Germination can occur after the release of dormancy and the occurrence of favourable environmental conditions. Although the hormonal control of seed dormancy is well studied, the molecular mechanisms underlying the induction and release of dormancy are not yet understood.

In this study, we report the cloning and characterization of the mutant *reduced dormancy 2-1* (*rdo2-1*). We found that *RDO2* is allelic to the recently identified dormancy gene *TFIIS*, which is a transcription elongation factor. *HUB1*, which was previously called *RDO4*, was identified in the same mutagenesis screen for reduced dormancy as *rdo2-1* and was also shown to be involved in transcription elongation. The human homologues of RDO2 and HUB1 interact with the RNA Polymerase II Associated Factor 1 Complex (PAF1C). Therefore, we investigated the effect of other Arabidopsis PAF1C related factors; *VIP4*, *VIP5*, *ELF7*, *ELF8* and *ATXR7* on seed dormancy. Mutations in these genes resulted in reduced dormancy, similar to *hub1-2* and *rdo2-1*. Consistent with a role at the end of seed maturation, we found that *HUB1*, *RDO2* and *VIP5* are upregulated during this developmental phase. Since mutants in PAF1C related factors are also described to be early flowering, we conclude that these components are involved in the regulation of both major developmental transitions in the plant.

## Introduction

Germination and induction of flowering are important developmental switches in the life cycle of plants. Seed dormancy is defined as the incapacity of a viable seed to germinate and evolved in plants to survive periods of unfavourable environmental conditions like dry summers. In many plant species, including the model plant *Arabidopsis thaliana*, primary seed dormancy is induced during the seed maturation phase and is highest in freshly harvested seeds. Dormancy is released by imbibition of seeds at low temperatures (stratification) or by dry storage (after-ripening). Germination requires the protrusion of the radicle through the surrounding structures (endosperm and testa in Arabidopsis) and can occur when non-dormant seeds meet permissive environmental conditions regarding humidity, light and temperature [1]. The depth of seed dormancy varies within and between plant species. Most important agricultural crop plants show shallow seed dormancy because this has been selected for during the domestication process. In some crops, including cereals, very low dormancy levels can lead to pre-harvest sprouting and consequently reduced product quality [2].

The plant hormone abscisic acid (ABA) is required for the induction of dormancy, whereas germination needs gibberellins (GA). Mutants that affect bioactive levels, or interfere with the signalling pathways of these hormones, usually show seed dormancy phenotypes [3], [4]. Several other hormones also influence dormancy and germination usually by interaction with ABA. Ethylene for instance acts antagonistically to ABA and promotes endosperm rupture [5]. Recently, a role for 12-oxo-phytodienoic acid (OPDA) in germination repression has been identified that is synergistic with ABA [6].

Despite the knowledge at the hormone level, the control of seed dormancy at the molecular level is still poorly understood. To obtain more insight in the molecular processes controlling dormancy, various mutagenesis screens and Quantitative Trait Locus (QTL) analyses have been performed. A major dormancy gene identified both by QTL analysis and mutagenesis screens is *DELAY OF GERMINATION 1* (*DOG1*). DOG1 encodes a protein with unknown function [7]. Another mutagenesis screen yielded four *reduced dormancy* (*rdo*) mutants in the Landsberg *erecta* genetic background [8], [9]. These mutants all have wild-type ABA levels and sensitivity and show only mild pleiotropic effects in the adult plant stage [9]. One of the underlying genes, *RDO4*, was cloned and renamed *HISTONE MONOUBIQUITINATION 1* (*HUB1*) [10]. *HUB1* encodes a C3HC4 RING finger protein, which is required for monoubiquitination of histone H2B. H2B ubiquitination regulates initiation and early elongation steps in transcription, whereas histone H2B deubiquitination is important for transcription elongation. It has been suggested that there might be multiple rounds of ubiquitination and deubiquitination during transcription elongation [11], [12]. Consistent with a role of histone ubiquitination in gene transcription efficiency, absence of functional HUB1 leads to altered expression of several dormancy-related genes [10].

Several other factors are known to play a role in transcription elongation, including Transcription factor S-II (TFIIS). TFIIS is able to overcome transcription arrest by RNA polymerase II and has recently been shown to control seed dormancy in Arabidopsis [13], [14]. In this work we demonstrate that the *rdo2* mutation, isolated in the same screen as *rdo4/hub1* [9], is allelic to *TFIIS*. This suggests that HUB1 and RDO2 both influence transcription efficiency. In agreement, a significant overlap in differentially expressed genes during seed maturation was found between both mutants. This confirms their involvement in the same process. Consistent with a role at the end of seed maturation, both genes are upregulated during this phase. The human homologues of RDO2 and HUB1 interact with the RNA Polymerase II Associated Factor 1 Complex (PAF1C) [14]. We show that mutants in several other PAF1C associated genes also have reduced dormancy. These mutants were originally isolated based on their early flowering phenotype. This indicates that PAF1C associated genes are involved in the regulation of both major developmental transitions in the plant.

## Results

### *RDO2* encodes a TFIIS transcription elongation factor

**Figure 1:**
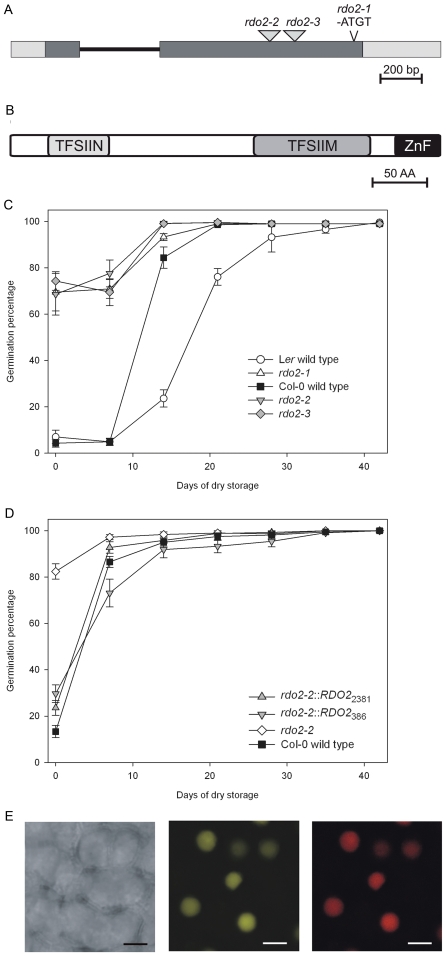
Characterisation of *RDO2*. (A) Schematic representation of the *RDO2* gene, indicating the positions of the *rdo2-1* 4 bp deletion and the *rdo2-2* and *rdo2-3* T-DNA insertions. Exons in the *RDO2* locus are represented as grey boxes, UTR regions as white boxes and the intron as a black line. (B) Schematic representation of RDO2 structural protein domains; Transcription factor IIS, N-terminal (TFSIIN), Transcription elongation factor S-IIM (TFSIIM) and Zinc finger (ZnF), obtained by At2g38560 protein analysis with the *Simple Modular Architecture Research Tool* (*SMART*) [49]. (C) Dormancy/germination behaviour of *rdo2-1* (white triangles up) and wild-type L*er* (white circles), *rdo2-2* (SALK_027259; grey triangles down), *rdo2-3* (SALK_133631; grey diamond) and wild-type Col (black squares). Germination is expressed as percentage of germinated seeds after different periods of seed dry storage starting from harvest. Error bars represent SE, n≥14. (D) Dormancy/germination behaviour of complemented *rdo2-2* mutants. The *rdo2-2* mutant (SALK_027259; white circles) was complemented with genomic-DNA fragments containing the complete *RDO2* coding sequence and 386 bp (*rdo2-2::RDO2_386_*; grey triangles down) or 2381 bp (*rdo2-2::RDO2_2381_*; grey triangles up) upstream of the *RDO2* start codon. Col wild-type is shown as black squares. Germination is expressed as percentage of germinated seeds after different times of seed dry storage starting from harvest. Error bars represent SE, n≥14. (E) YFP signal in nuclei of *rdo2* mutant plants that were stably transformed with a p2X35S:RDO2:YFP construct. Left panel transmission, middle panel YFP fluorescence, right panel Propidium Iodide staining. Scale bar represents 3 µm.

We aimed to identify the *rdo2* mutation, which causes reduced dormancy and maps at the bottom of chromosome 2 [9]. To reduce the influence of natural variation between different accessions and to ease the recognition of the mutant phenotype during the mapping process, *rdo2-1* was crossed with the Near Isogenic Line (NIL) LCN2-18 [15]. This NIL has a L*er* isogenic genetic background, except for a 4.5 Mb introgression of Cvi at the bottom of chromosome 2 containing the *RDO2* locus. Using a mapping population of 1100 F2 plants, the location of *rdo2-1* could be assigned to a region of 46 kb between the markers T6A23-1 and T6A23-2 located at respectively 16.123 and 16.169 Mb. This region contains 15 annotated genes. Based on the structure of these genes (analyzed in The Arabidopsis Information Resource [16]) and their expression pattern (analyzed with Genevestigator [17]), the candidate gene At2g38560 was selected. Sequencing revealed a four bp deletion at the end of the coding sequence of At2g38560 in the *rdo2-1* mutant (Figure 1A). The protein encoded by this gene contains three structural domains, named Transcription factor IIS N-terminal (TFSIIN), Transcription elongation factor S-IIM (TFSIIM) and Zinc finger (ZnF) (Figure 1B). This combination of domains is characteristic for Transcription elongation factor SII (TFIIS) [18]. Due to the 4 bp deletion, the *rdo2-1* mutant gene translates into a protein lacking the ZnF domain, which most likely renders it not functional. At2g38560 has previously been identified as a dormancy gene by Grasser and colleagues [13], who named the gene TFIIS.

The identity of At2g38560 as *RDO2* was confirmed with additional independent T-DNA insertion mutant alleles (*rdo2-2/tfIIs-2* [13] and *rdo2-3*; Figure 1A) in the Columbia (Col) background. Both insertion mutants lack full-length *RDO2* mRNA and showed reduced dormancy, similar to *rdo2-1* (Figure 1C). In addition, complementation of *rdo2-2* with the *RDO2* genomic locus complemented the mutant phenotype (Figure 1D).

*RDO2* is ubiquitously expressed throughout all plant tissues as shown in the Arabidopsis eFP browser [19] and [13]. These authors also showed that the Arabidopsis TFIIS protein is localized in the nucleus of transiently transformed protoplasts, which is consistent with a role for RDO2 in transcription elongation [13]. In agreement, we detected YFP signal in nuclei of *rdo2-1* mutant plants that were stably transformed with a p2X35S:RDO2:YFP construct (Figure 1E). However, this construct did not complement the *rdo2-1* phenotype. This indicates that the YFP tag probably interferes with RDO2 function.

### Mutations in *HUB1* and *RDO2* affect the expression of an overlapping set of genes

**Figure 2:**
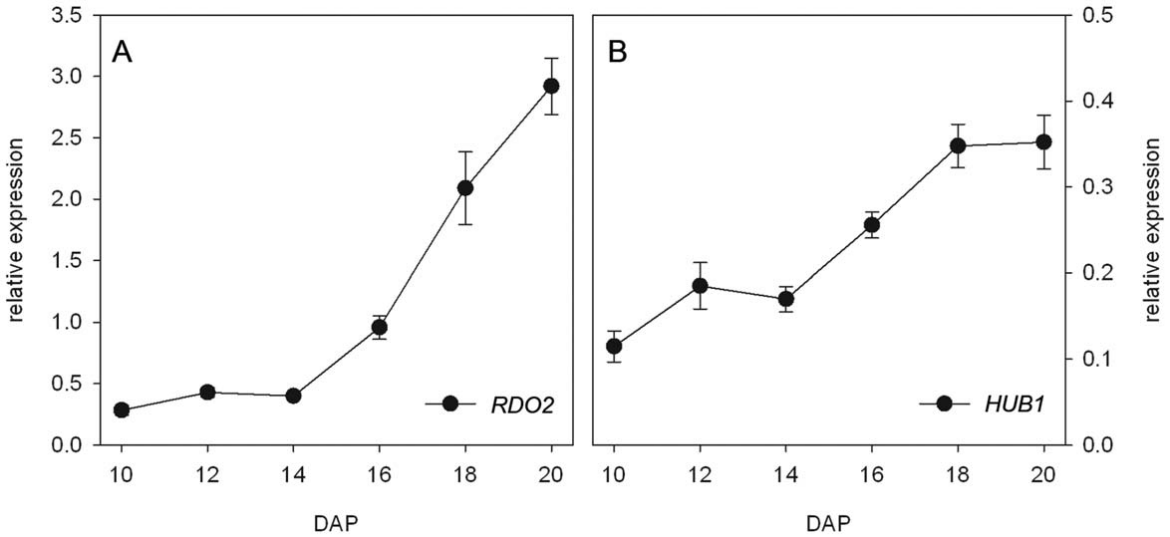
*HUB1* and *RDO2* transcription during seed maturation. (A–B) Relative expression of (A) *RDO2* and (B) *HUB1* in L*er* during seed maturation at 10–20 days after pollination (DAP) compared to *ACTIN8* expression. Error bars represent SE, n≥3.

**Figure 3:**
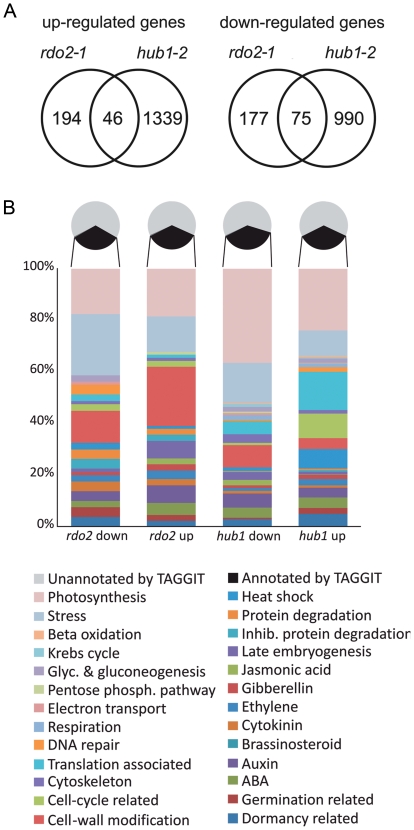
Transcriptome analysis of *rdo2-1* and *hub1-2* siliques at 18–19 DAP. (A) Venn diagram showing the number of overlapping and unique up- and downregulated genes in *rdo2*-*1* and *hub1-2*. (B) *TAGGIT* gene ontology classification of up- and downregulated genes in *rdo2*-*1* and *hub1-2*.

The *hub1-2* mutant (previously named *rdo4*) was identified in the same mutagenesis screen as *rdo2-1* [9]. *HUB1* is required for monoubiquitination of histone H2B [10]. This histone modification is involved in transcription initiation and elongation [12], which suggests that RDO2 and HUB1 are involved in the same process. The *RDO2* and *HUB1* genes are both ubiquitously expressed. Because their mutants show reduced seed dormancy, we analyzed their expression dynamics in detail during seed maturation. RT-PCR analysis indicated that both genes are strongly upregulated during this phase (Figure 2A, B). This increase in expression levels, together with the identity of HUB1 and RDO2 as transcription initiation and elongation factors and the observation that *rdo2-1* and *hub1-2* mutants have reduced dormancy levels, indicates that transcription maintenance towards the end of seed maturation is probably required for the induction of seed dormancy. Therefore, we analyzed the transcriptomes of nearly ripe siliques (18–19 DAP) of the *hub1-2* and *rdo2-1* mutants in comparison with wild-type L*er* using Affymetrix GeneChip Arabidopsis ATH1 Genome Micro-Arrays. The *hub1-2* and *rdo2-1* mutants revealed respectively 2450 and 492 differentially expressed genes (Benjamini & Hochberg (BH) adjusted P-value<0.01) (Dataset S1). The *hub1-2* mutant thus has a stronger influence on the transcriptome than *rdo2-1*. A relatively high number of differentially expressed genes (46 up- and 75 downregulated) overlapped between both mutants (Figure 3A). The significance of this overlap was determined by calculating the representation factor, which is the number of overlapping genes divided by the expected number of overlapping genes drawn from two independent random picked groups [20]. The representation factor for upregulated genes in *rdo2-1* and *hub1-2* is 3.1 (p<3.243e-12) and for downregulated genes 6.4 (p<1.268e-39). HUB1 and RDO2 are both positive regulators of transcription and direct targets of these proteins are expected to be found among the downregulated genes. The downregulated genes indeed showed the highest overlap between *hub1-2* and *rdo2-1* (30% of the total number of downregulated genes in *rdo2-1* overlaps with *hub1-2*, compared to 19% overlap for upregulated genes). One of the downregulated genes in both *hub1-2* and *rdo2-1* is the dormancy gene *DOG1* (Figure S1). DOG1 protein is required for the induction of dormancy and differences in *DOG1* expression can explain differences in dormancy levels [7]. Therefore, downregulation of *DOG1* likely contributes to the reduced dormancy of *hub1-2* and *rdo2-1*.

We used the seed-specific gene ontology classification, called *TAGGIT* [21], to analyze the differentially regulated genes (Figure 3B). Stress related genes are mainly found among the down-regulated genes of both mutants. The *rdo2-1* transcriptome is characterized by an upregulation of cell-wall modifying and late-embryogenesis genes. The *hub1-2* transcriptome shows an upregulation of translation associated, cell-cycle related and heat shock genes.

### Predicted PAF1C associated factors are upregulated during seed maturation and are required for the induction of seed dormancy

**Figure 4:**
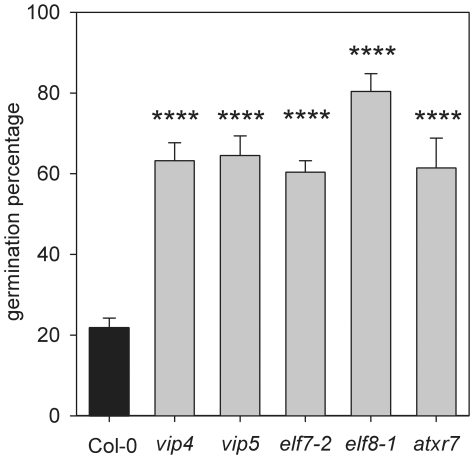
RNAPII associated factors are required to establish dormancy. Dormancy/germination behaviour of freshly harvested seeds of Col wild-type (black bar), *vip4*, *vip5*, *elf7-2*, *elf8-1* and *atxr7-1* (gray bars). Error bars represent SE, n≥10, except *elf8-1* n = 3; Error bars represent SE. Significance levels: ****p<0.0001; 2-tailed Student's T-test, compared to Col-0.

**Figure 5:**
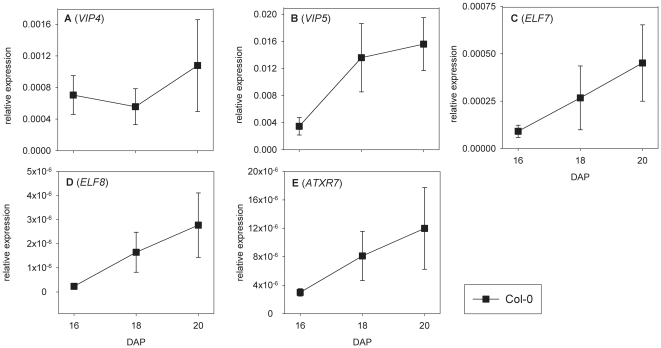
PAFIC associated factors are upregulated during seed maturation. Transcript abundance of (A) *VIP4*, (B) *VIP5*, (C) *ELF7*, (D) *ELF8*, (E) *ATXR7*, during seed maturation at 16–20 days after pollination (DAP) compared to *ACTIN8* expression. Error bars represent SE, n = 4.

The human homologues of HUB1 and RDO2 are Bre1 and TFIIS respectively. Both interact with the human RNA Polymerase II Associated Factor 1 Complex (PAF1C) [14], [29], which provides a platform for the association of complexes that modulate the structure of chromatin during transcription elongation [30]. Accordingly, PAF1C has a crucial role in the regulation of histone monoubiquitination and is required for recruitment of Set1 and Set2 proteins. These proteins are involved in methylation of histone H3 at respectively K4 and K36, which are activating epigenetic marks for transcription [30]. VERNALIZATION INDEPENDENCE 4 (VIP4), VIP5, EARLY FLOWERING 7 (ELF7) and ELF8 are the Arabidopsis homologues of respectively the yeast proteins Leo1, Rtf1, PAF1 and CTR9, which are all components of PAF1C [31], [32], [33]. *ARABIDOPSIS TRITHORAX-RELATED 7* (*ATXR7*) is the Arabidopsis homologue of Set1 [34]. As shown in Figure 4, the *vip4*, *vip5*, *elf7-2*, *elf8-1* and *atxr7* mutants all show significantly (*p<*0.0001) reduced seed dormancy levels, similar to *rdo2-1* and *hub1-2*. Alike *HUB1* and *RDO2*, most of these genes also show a tendency towards upregulation at the end of seed maturation between 16 and 20 DAP (Figure 5), which is however only significant for *VIP5* (p = 0.0255) (Figure 5B).

**Figure 6:**
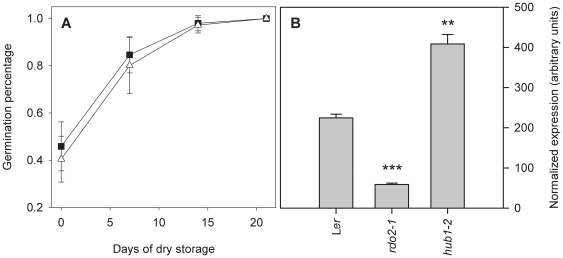
FLC does not affect seed dormancy. (A) Dormancy/germination behaviour of Col (black squares) and *flc101* (open triangles). Germination is expressed as percentage of germinated seeds after different times of seed dry storage. Error bars represent SE, n = 15. (B) *FLC* expression in siliques, 18–19 days after pollination (DAP) of wild-type L*er* and the *rdo2-1* and *hub1-2* mutants. Expression data were obtained from the microarray experiment described in this study. Significance levels: **p<0.01, ***p<0.001; 2-tailed Student's T-test, compared to wild type L*er*.

VIP4, VIP5, ELF7, ELF8 and ATXR7 are all required for expression of the flowering repressor *FLC* [28] and their corresponding mutants are early flowering [32], [33], [35]. In addition, *FLC* expression is decreased in the *hub1-4* mutant and *hub1* and *tfIIs* mutants are early flowering [13], [22], [23]. FLC may therefore represent a connection between the regulation of flowering time and seed dormancy as it is expressed in seeds and has been shown to promote germination at low temperatures [36]. However, we could not detect an altered seed dormancy phenotype in the *flc* mutant (Figure 6A). Moreover, our microarray data indicate that *FLC* expression in mature siliques is 3-times lower in *rdo2-1*, but 2-times higher in *hub1-2* compared to the wild-type (Figure 6B). Therefore, it is not likely that the reduced dormancy phenotype of the studied mutants is caused by altered *FLC* expression.

## Discussion

The plant's life cycle is controlled by the timing of two major developmental transitions, germination and induction of flowering. The molecular pathways that control flowering and its interaction with the environment are well studied [37]. However, the control of germination by seed dormancy is still poorly understood at the molecular level. A mutagenesis screen for reduced dormancy in the L*er* background yielded four mutants [9], of which the first, *hub1-2*, was recently cloned [10]. Here, we report the cloning and characterization of a second mutant, *rdo2-1*. Interestingly, both identified genes are predicted to function in transcription elongation and to associate with the PAF1 complex. Accordingly, transcriptome analysis showed a highly significant overlap in the differentially expressed genes of both mutants. This motivated us to analyse the germination behaviour of additional mutants in genes related to PAF1C, which indeed all showed reduced seed dormancy.

### *RDO2* encodes a *TFIIS* transcription elongation factor

The *rdo2* mutant was isolated based on its reduced seed dormancy, but also shows some mild additional phenotypes including earlier flowering [8], [13]. The *rdo2-1* mutation consists of a 4 bp deletion in a gene encoding a protein with high homology to yeast and human TFIIS (Figure 1B). Originally, TFIIS was isolated as a factor that can stimulate RNA synthesis by specifically stimulating RNAPII [38]. It has been reported that the Arabidopsis TFIIS gene can partially complement the yeast *tfIIs* mutant [13], indicating that Arabidopsis *RDO2* is a *bona fide* TFIIS transcription elongation factor.

Gene transcription is not only controlled by the recruitment of RNAPII to the promoter, but also by the elongation speed of the moving RNAPII along the coding strand [30]. RNAPII can become paused during elongation at certain sites, which is likely determined by the strength of histone-DNA contacts. This pausing leads to backtracking of the RNAPII, which can cause a complete arrest of transcription [39]. TFIIS helps to overcome such an arrest by stimulating a cryptic, nascent RNA cleavage activity intrinsic to RNAPII [18], [30]. PAF1 is an evolutionary conserved elongation factor complex that was shown to affect transcription elongation efficiency by H2B ubiquitination and H3K4 and H3K79 methylation. It was recently shown that PAF1C also has a more direct role in transcription elongation by its direct interaction with TFIIS and cooperative binding to RNAPII in human HeLa cells [14].

The Arabidopsis TFIIS gene has previously been described as a dormancy gene by Grasser and colleagues [13]. However, in their work increased germination could only be observed after removal of immature seeds at 15 DAP from siliques and freshly harvested seeds germinated at equal rates in the wild-type and *tfIIs* mutants. In contrast to this, we observed a clear dormancy phenotype in freshly harvested seeds for all three studied *rdo2* mutant alleles (Figure 1C). One of these mutants, *rdo2-2*, is identical to *tfIIs-2*, for which dormancy phenotype was detected previously in freshly harvested seeds [13]. The different dormancy phenotypes could be explained by differences in the growth conditions because dormancy levels are strongly influenced by the environment.

### The influence of *RDO2* and *HUB1* on gene transcription in maturing seeds

RDO2 and HUB1 are both predicted to influence gene transcription. Our transcriptome analysis showed that indeed relatively high numbers of genes are differentially expressed in the *rdo2-1* and *hub1-2* mutants (Figure 3A). The number of differentially expressed genes is about five times higher in the *hub1-2* mutant compared to the *rdo2-1* mutant. This indicates that absence of histone H2B ubiquitination has a stronger impact on gene expression than absence of TFIIS dependent transcription elongation. RDO2 and HUB1 are both positive regulators of transcription and direct target genes of these factors are therefore expected to be down-regulated in the *rdo2-1* and *hub1-2* mutants. Surprisingly, the number of up-regulated genes is similar to the number of down-regulated genes for both *rdo2-1* and *hub1-2* (Figure 3A). This indicates that a high number of the differentially expressed genes are probably indirect targets of RDO2 and HUB1. In contrast, a higher number of genes down-regulated was found in the *tfIIs* mutant compared to the number of up-regulated genes [13]. This difference with our transcriptome analysis could be explained by the different material that was used for the experiments. Grasser and colleagues analysed seedlings for their transcriptome analysis [13], whereas we used siliques at 18–19 DAP. Interestingly, we found an upregulation of the flowering repressor gene *FLC* in siliques of *hub1*, whereas earlier studies showed a downregulation of *FLC* in *hub1* seedlings [22], [23]. This underlines that the influence of HUB1 on transcription is different between seeds and seedlings.

We found a relatively high number of down-regulated ‘stress related’ genes in both *rdo2-1* and *hub1-2* (Figure 3B). Genes belonging to this TAGGIT class are normally upregulated during seed maturation, probably due to the stress conditions caused by desiccation of the maturing seed [24]. These genes could be direct targets of RDO2 and HUB1 and might require these elongation factors to obtain sufficiently high expression levels towards the end of seed maturation. Despite the reduced expression levels of stress related genes in *rdo2-1* and *hub1-2* mutants, we have not observed any obvious stress related phenotype under our growth conditions. The only clear seed phenotype in *rdo2-1* and *hub1-2* mutants is reduced dormancy, which can be partially explained by down-regulation of *DOG1* in both mutants (Figure S1).

The *rdo2-1* transcriptome is characterized by an upregulation of cell-wall modifying and late-embryogenesis genes. The *hub1-2* transcriptome shows an upregulation of translation associated, cell-cycle related and heat shock genes (Figure 3B). Genes that regulate cell-wall modification, translation and cell-cycle are upregulated during after-ripening in wild type seeds, probably causing an increased germination potential [25]. Therefore, upregulation of these genes in *rdo2-1* and *hub1-2* could be contributing to the reduced dormancy of these mutants. In contrast, late-embryogenesis and heat shock genes are associated with dormant expression patterns [25] and their increased expression in *rdo2* and *hub1* indicates that they are probably independent of the reduced dormancy phenotype.

### PAF1C associated factors control both germination and flowering time

*RDO2* is a single copy gene in Arabidopsis and highly conserved among eukaryotes. Despite its high conservation and its role in an essential process, the mutant phenotype is weak in Arabidopsis. Weak mutant phenotypes for TFIIS are found in more eukaryotes. *S. cerevisiae* TFIIS null mutants for instance only show sensitivity to 6-azauracil [26]. In contrast, mice that lack TFIIS die during embryo development at the mid-gestation phase [27]. Similar to *rdo2* mutants, the Arabidopsis mutants *hub1*, *elf7-2*, *elf8-1*, *vip4*, *vip5* and *atxr7* all show no, or weak, pleiotropic phenotypes [28], [31], [32], [35]. The lack of a strong phenotype for all these mutants is probably due to the presence of multiple elongation factors in eukaryotic cells that function both cooperatively and redundantly [30]. However, this redundancy does not completely compensate for negative effects on transcription of genes required for flowering time and dormancy. Alternatively, these PAF1C associated proteins are only required for transcription of a subset of all genes.

Our data suggest that PAF1C associated factors are required to facilitate expression during late seed maturation, since genes encoding predicted PAF1C associated factors showed a trend towards increased expression during seed maturation (Figures 2 and 6). It is unlikely that increased gene expression at the end of seed maturation is a general phenomenon, as it has been shown that the end of seed maturation is characterized by decreased metabolic activities, including gene transcription [40].

The upregulation of PAF1C associated genes at the end of seed maturation and the clearly reduced dormancy phenotypes of their mutants indicate that they might be especially important in this phase, possibly by counteracting negative effects of desiccation on gene expression.

### Conclusion

Overall, our data indicate that PAF1C associated factors are involved in both the control of flowering time and dormancy/germination. They regulate flowering by controlling *FLC* expression and dormancy by control of the expression of yet unidentified genes. FLC is a flowering repressor that is downregulated by vernalisation and its expression has to be reset every generation. It has been shown that *FLC* expression is reactivated during embryogenesis [41], [42]. Therefore, the PAF1C associated factors probably control the expression of *FLC* and dormancy genes simultaneously during seed development. A role for PAF1C associated genes, including the here reported *RDO2* gene, as factors regulating both flowering time and seed dormancy could have ecological implications. The moment when a seed germinates will determine the environmental conditions (especially daylength and temperature) to which the plant will be exposed during further growth and thereby indirectly influences life-history traits, including flowering time [43]. Factors like RDO2 and HUB1 could therefore be part of a mechanism that links the germination time to the flowering time, in order to obtain maximum fitness.

## Materials and Methods

### Plant materials and growth conditions

The *rdo2-1* (L*er*) mutant is described by [9], *hub1-2* (L*er*) by [10], *vip4* (Col) by [31] and *vip5* (Col) by [33]. The *elf7-2* and *elf8-1* (Col) mutants [32] and LCN2-18 [15] were kind gifts of the authors who described the lines. The *rdo2-2*/*tfIIs-2* [13] (SALK_027259), *rdo2-3* (SALK_133631) and *atxr7-1* (SALK_14691c) mutants were obtained from the SALK T-DNA insert collection [44]. The *rdo2-2* mutant contained a T-DNA insert at 1073 bp, *rdo2-3* at 1195 bp and *atxr7-1* at 782 bp downstream of the start codon. Full-length mRNAs of the respective genes could not be detected in these mutants. For complementation analysis, the L*er RDO2* genomic locus (including 386 or 2381 bp upstream of the *RDO2* start codon) was cloned into the vector pGW-MCS_nos and stably transformed into *rdo2-2*. Confocal microscopy (Leica TCS SP2, Germany) was used to detect the YFP signal in *rdo2-1* mutant plants that were stably transformed with a p2X35S:RDO2:YFP construct generated using the pENSG-YFP [45] vector. Propidium Iodide was used for counterstaining.

Plants were grown on soil containing a mixture of substrate and vermiculite (3∶1). Plants for the germination tests and transcript analyses were grown in Elbanton growth cabinets (Elbanton BV, Kerkdriel, the Netherlands) in long day conditions (16 h light at 22°C and 8 h dark at 16°C). Plants for mapping and crossings were grown in an air-conditioned greenhouse with a day temperature of 20°C and a night temperature of 18°C; 16 h of light was provided daily.

### Germination tests

Approximately 50 seeds of individually harvested plants were sown on filter paper, put into transparent moisturized containers and incubated in a germination cabinet (Van den Berg Klimaattechniek, Montfoort, the Netherlands) in long-day conditions (16 h light at 25°C, followed by 8 h darkness at 20°C). After 7 d of incubation, the germination percentages were analyzed with a ‘Germinator’ setup and analyzed as described in [46]. After-ripening conditions of dry seed batches occurred in darkness at 21°C, 50% RH in a controlled cabinet (MMM Medcenter, Brno, Czech Republic).

### Transcriptomics

Extraction of RNA from 18–19 DAP siliques was performed using RNAqueous columns (Ambion, Austin, TX, USA). Affymetrix GeneChip Arabidopsis ATH1 Genome Array microarray hybridization and subsequent analysis was performed in house. For all microarray experiments, RNA from three independent biological replicates was used for hybridization and subsequent analyses. Processing and statistical analysis of the microarray data were done in Expressionist Pro v5.1 (Genedata AG, Basel, Switzerland). We used the GC-RMA algorithm for background correction, normalization and probe summarization. Various quality metrics were examined to exclude quality problems. All microarray data are MIAME compliant and have been deposited at the Gene Expression Omnibus database (GEO Accession number: GSE28446).

Control probe sets and probe sets with MAS5 detection P-value smaller than 0.05 in less than two of the nine arrays were filtered out prior to further analysis, leaving 14,655 probe sets. Differential expression of genes (Dataset S1) between each mutant and L*er* was assessed using the regularized Bayesian T-test CyberT [47], [48]. BH adjusted P-value (false discovery rate) was adopted for correction of multiple testing [48]. BH adjusted P-value<0.01 was taken as criteria for differential expression.

For the statistical significance of the overlap between two groups of genes we used a web-based tool at http://elegans.uky.edu/MA/progs/overlap_stats.html. We used the seed-specific gene ontology classification program, called TAGGIT [21], to analyze the differentially regulated genes.

### Q-RT PCR

RNA from seeds was extracted using RNAqueous small scale Phenol-free total RNA isolation kit in addition with RNA isolation aid (Ambion, Austing, TX, USA). After elution (95°C warm elution buffer), the RNA was cleaned via a high salt precipitation (1.2 M Tri-Na citrate-dihydrate +0.8 M NaCl), washed with 70% ethanol, dried and dissolved. Thereafter, the RNA was precipitated using 25 M LiCl, rinsed with 2 M LiCl, washed with 70% ethanol, dried and dissolved. cDNA synthesis was proceeded with QuantiTect Reverse Transcription Kit (Qiagen, Cat No.205311) including DNAse treatment (gDNA wipeout buffer). Quantitative RT-PCR was subsequently performed via standard procedures using QuantiTect SYBR Green PCR Kit (Qiagen, Cat No.204143) on an Eppendorf Mastercycler realplex^2^, epgradient cycler. Expression was calculated relative to *ACT8* (AT1G49240). All primers used in this study can be found in Table S1. All primers were BLAST searched against the Arabidopsis genome to check uniqueness.

## Supporting Information

Figure S1 ***DOG1*** **is downregulated in** ***rdo2-1*** **and** ***hub1-2*** **.** *DOG1* expression in siliques, 18–19 days after pollination (DAP) of wild-type L*er* and the *rdo2-1* and *hub1-2* mutants. Expression data were obtained from the microarray experiment described in this study. Significance levels: *p<0.05 **p<0.01; 2-tailed Student's T-test, compared to wild type L*er*.
(TIF)

Table S1 **Primer combinations used for RT-PCR analysis.**
(DOC)

Dataset S1 **Differentially expressed genes in the** ***hub1-2*** **and** ***rdo2-1*** **mutants.**
(XLS)
