# Supplementary figures and images for: Identification of the Arabidopsis REDUCED DORMANCY 2 Gene Uncovers a Role for the Polymerase Associated Factor 1 Complex in Seed Dormancy
Source: PLoS One. 2011 Jul 25;6(7):e22241. doi: 10.1371/journal.pone.0022241 (PMC3143138; doi:10.1371/journal.pone.0022241)

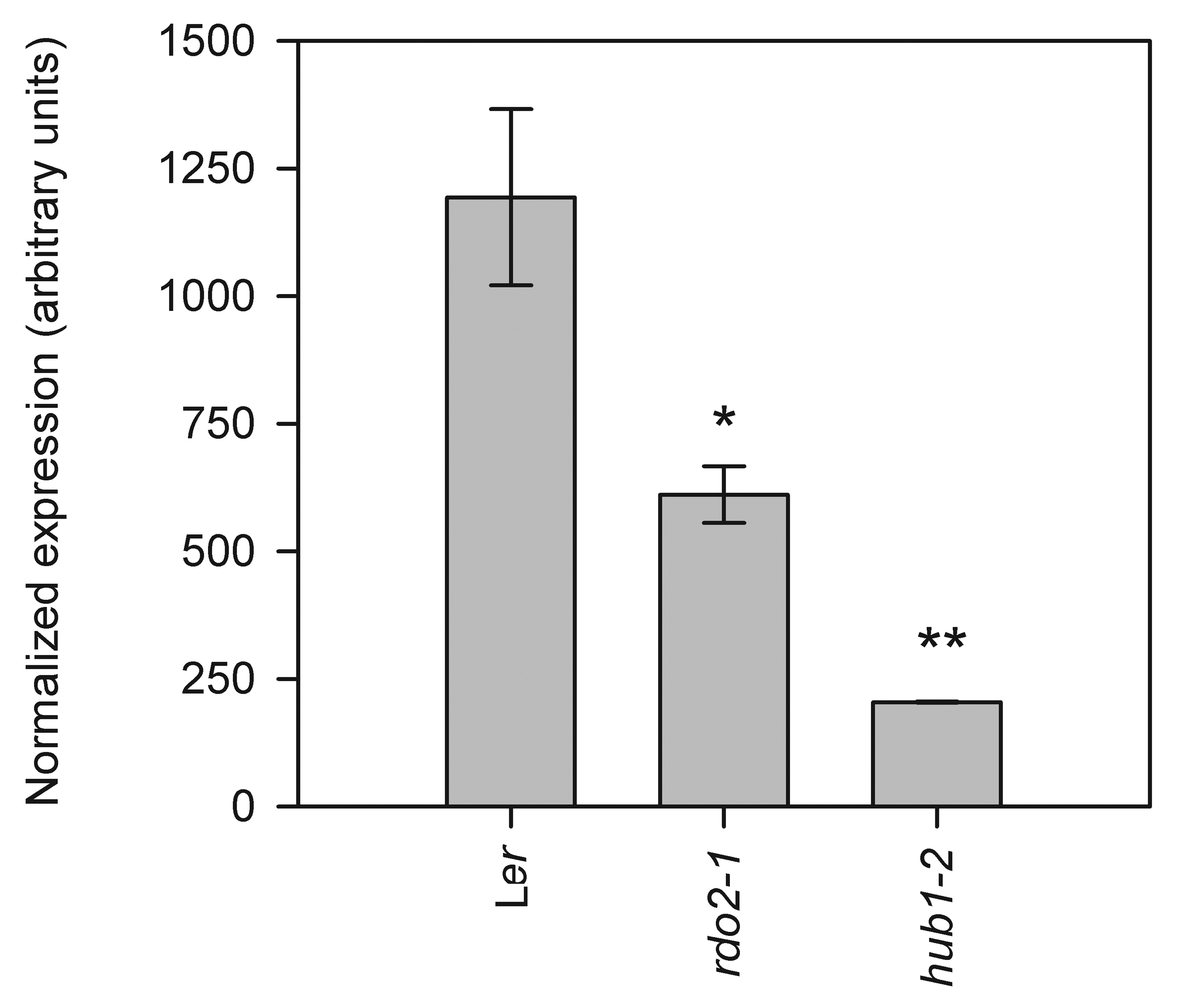

Supplement: Figure S1 — DOG1 is downregulated in rdo2-1 and hub1-2 . DOG1 expression in siliques, 18–19 days after pollination (DAP) of wild-type Ler and the rdo2-1 and hub1-2 mutants. Expression data were obtained from the microarray experiment described in this study. Significance levels: *p<0.05 **p<0.01; 2-tailed Student's T-test, compared to wild type Ler. (TIF) [file pone.0022241.s001.tif]
