# Supplementary material for: Identification of the Arabidopsis REDUCED DORMANCY 2 Gene Uncovers a Role for the Polymerase Associated Factor 1 Complex in Seed Dormancy
Source: PLoS One. 2011 Jul 25;6(7):e22241. doi: 10.1371/journal.pone.0022241 (PMC3143138; doi:10.1371/journal.pone.0022241)
Supplement: Table S1 — Primer combinations used for RT-PCR analysis. (DOC) [file pone.0022241.s002.doc]

**Table SI. Primer combinations used for RT-PCR analyis.**

*ACT8*: 5’-CTCAGGTATTGCAGACCGTATGAG-3’ 5’-CTGGACCTGCTTCATCATACTCTG-3’

*RDO2*: 5’-CCACTGGAAGTTCTGTTGAGG-3’ 5’-CTGCTAGCAAATGGACACGA-3’

*HUB1*: 5’-TGGGGCATTAGAACTGGAAC-3’ 5’-GGCCGATGATCCTTCTATGA-3’

*VIP4:* 5’-GAAGAGCCACAAAGGCATTC-3’ 5’-ATCAATCACGGCCTTACGAC-3’

*VIP5:* 5’- GATGGACCCGATCATCAAAC-3’ 5’- CTTCGGTCAGTTGCTTCCTC-3’

*ELF7:* 5’- TGGTTCCTTCCTTGGATGAG-3’ 5’- CGAAGCACCAACCCTAGAAG-3’

*ELF8:* 5’- CCAACATGTATGCTGCCAAC-3’ 5’- TTTGCATTACAGCACCCAAA-3’

*ATXR7:* 5’- TCATGTCGCTACTGGCTTTG-3’ 5’- CATGCTGCTGCCAACTAAAA-3’
